# Boosting spin-caloritronic effects by attractive correlations in molecular junctions

**DOI:** 10.1038/srep19236

**Published:** 2016-01-25

**Authors:** Ireneusz Weymann

**Affiliations:** 1 Faculty of Physics, Adam Mickiewicz University, ul. Umultowska 85, 61-614 Poznań, Poland

## Abstract

In nanoscopic systems quantum confinement and interference can lead to an enhancement of thermoelectric properties as compared to conventional bulk materials. For nanostructures, such as molecules or quantum dots coupled to external leads, the thermoelectric figure of merit can reach or even exceed unity. Moreover, in the presence of external magnetic field or when the leads are ferromagnetic, an applied temperature gradient can generate a spin voltage and an associated spin current flow in the system, which makes such nanostructures particularly interesting for future thermoelectric applications. In this study, by using the numerical renormalization group method, we examine the spin-dependent thermoelectric transport properties of a molecular junction involving an orbital level with attractive Coulomb correlations coupled to ferromagnetic leads. We analyze how attractive correlations affect the spin-resolved transport properties of the system and find a nontrivial dependence of the conductance and tunnel magnetoresistance on the strength and sign of those correlations. We also demonstrate that attractive correlations can lead to an enhancement of the spin thermopower and the figure of merit, which can be controlled by a gate voltage.

Thermoelectric transport properties of nanoscale devices have recently attracted a considerable attention^1^,^2^,^3^,^4^. In nanostructures, such as e.g. quantum dots or molecules coupled to external leads, the thermoelectric response of the system to applied temperature gradient 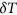 can be enhanced compared to conventional bulk materials, which is due to the size quantization and interference effects^5^,^6^,^7^,^8^,^9^. This can result in large values of the Seebeck coefficient *S* and the corresponding figure of merit 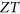, which can take values close to or even exceeding unity. This consequently makes such nanosystems promising from the application point of view. For example, huge figure of merit due to quantum interference was recently predicted in artificial molecules built of coupled quantum dots^10^,^11^,^12^,^13^ Moreover, an enhancement of thermoelectric properties was also found for the single-level molecules exhibiting attractive Coulomb interactions, 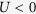
14. Such attractive interactions can be induced e.g. by coupling to phonons or due to vibronic modes in a molecular junction^15^,^16^.

When a temperature gradient is applied to magnetic nanostructures, the thermoelectric response of the system becomes spin-dependent^17^. In other words, 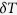 can generate a spin-dependent voltage drop, which can then give rise a flow of spin current in the system^18^. For this reason, spin caloritronic properties of various systems have recently been extensively studied^19^,^20^,^21^. Especially interesting in this regard are quantum dots and molecules, in which quantum confinement can lead to large spin-resolved thermoelectric response^22^,^23^,^24^,^25^,^26^,^27^,^28^,^29^,^30^,^31^,^32^. The existing considerations involved both the weak coupling regime, where the single-electron charging effects are relevant^33^, as well as the strong coupling regime, when electron correlations lead to the Kondo phenomenon^34^. Due to the Kondo effect, the conductance through the system increases to its maximum value of 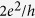 for temperatures *T* smaller than the Kondo temperature 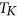
34. For nonmagnetic systems, the Kondo correlations are revealed through a sign change of the thermopower as a function of temperature^35^. However, both the Seebeck coefficient and the figure of merit are then not particularly large. Because the thermoelectric properties were shown to be enhanced in the case of attractive correlations in the molecule^14^, here we extend these studies onto the spin-resolved thermo-transport properties.

We thus address the problem of spin-caloritronic properties of a molecular junction with ferromagnetic contacts, focusing on the Kondo regime. To model such system we use the negative-*U* Anderson model^14^,^36^ and employ the numerical renormalization group (NRG) method^37^. First, we analyze how the spin-resolved electronic properties of the system change when attractive correlations emerge in the system. Then, we study the spin caloritronic properties of the device, such as the (spin) Seebeck coefficient and the corresponding figure of merit, in the case of both fast spin relaxation in the leads and in the absence of spin relaxation when the spin accumulation builds up. By directly comparing results obtained for the negative-*U* model with the positive-*U* case, we find a large enhancement of spin-resolved thermoelectric properties of the system caused by attractive correlations in the molecule. An increase of the figure of merit occurs both in the presence of finite spin accumulation in the leads and in the case when the spin relaxation is fast.

## Results

The considered system consists of a molecule coupled to ferromagnetic leads whose magnetic moments can be oriented either in parallel or in antiparallel. The molecule is modeled by a single orbital level of energy *ε* and Coulomb correlations *U*, which is coupled to the left 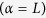 and right 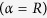 lead with the coupling strength 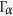
36. In the case of finite spin polarization of the leads 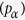 the couplings are spin dependent. In the parallel configuration one then has, 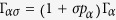, while in the antiparallel configuration the coupling to the right lead changes and is given by, 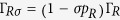. In the following, we assume that the system is left-right symmetric, 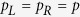, and 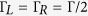. As energy unit we will use the band halfwidth 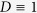.

In this analysis we in particular focus on molecules, in which the Coulomb correlations can become attractive, 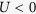
14,15,16. Attractive correlations can result e.g. from vibrations of the molecule or from strong electron-phonon coupling. We will, however, not consider any particular mechanism responsible for such attractive correlations, but will rather focus on analyzing and discussing general effects of negative-*U* on spin-resolved electric and thermoelectric transport behavior. To study the transport properties of the considered molecular junction, we employ the full density-matrix numerical renormalization group (NRG) method^37^,^38^. By using NRG, we are able to accurately analyze the linear-response transport in the Kondo regime. Here, we are particularly interested in the spin-dependent conductance, thermopower and thermoelectric figure of merit. First, we analyze how the emergence of attractive correlations affects the linear response conductance in both magnetic configurations and the resulting tunnel magnetoresistance (TMR) and then study the spin caloritronic effects.

### Magnetoresistive properties

**Figure 1:**
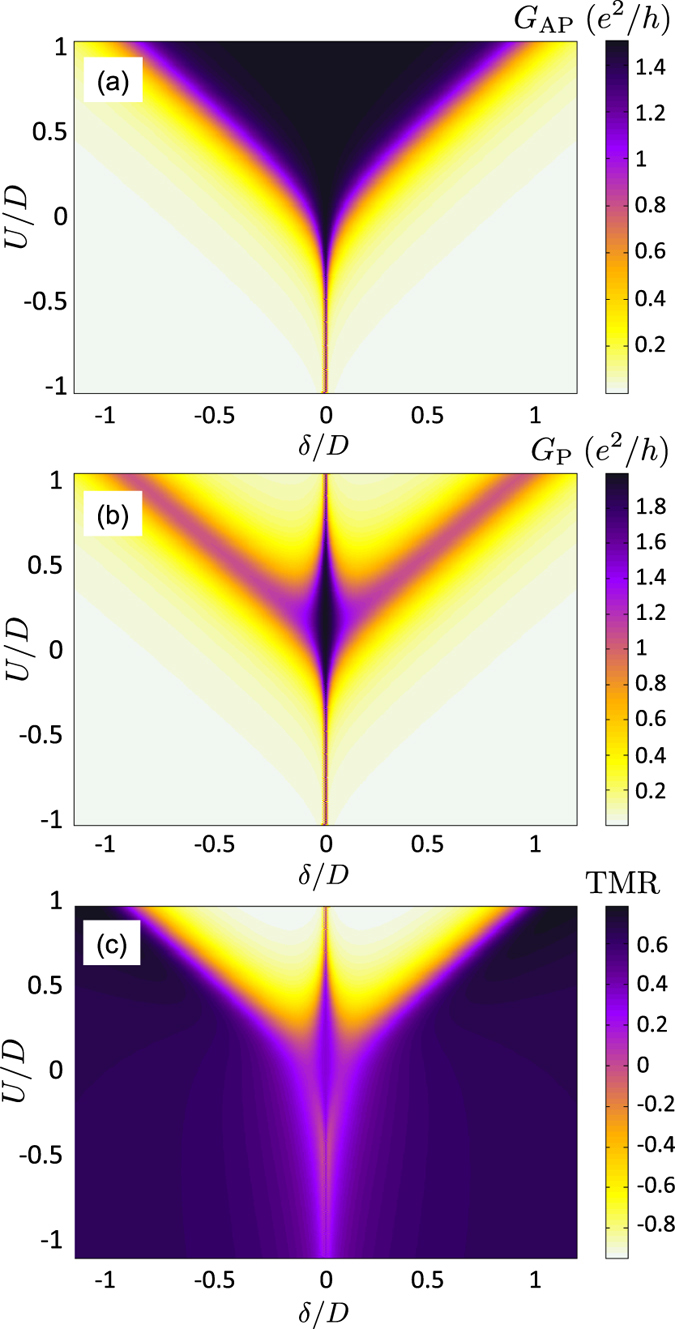
Spin-resolved transport properties. The linear conductance in (**a**) the antiparallel and (**b**) parallel magnetic configuration and (**c**) the resulting TMR as a function of the level detuning 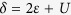 and the Coulomb correlation parameter *U*. The parameters are: 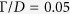, 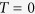 and 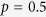. *D* denotes the band halfwidth, which is used as energy unit, 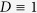.

The dependence of the zero-temperature linear-response conductance in the parallel 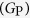 and antiparallel 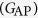 magnetic configurations and the resulting TMR on the detuning from the particle-hole symmetry point 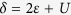 and Coulomb correlation parameter *U* is shown in Fig. 1. Here, the tunnel magnetoresistance is defined as^39^ 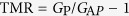. This figure effectively demonstrates how the transport properties change when crossing over from the positive-*U* to the negative-*U* case. In the antiparallel configuration, for 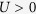 and 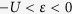, the conductance exhibits a plateau due to the spin Kondo effect with 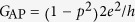
40,41. This behavior, except for spin polarization dependent factor, is equivalent to nonmagnetic systems, as long as the left-right symmetry is not broken^42^. When the Coulomb correlations become attractive, the empty and doubly occupied states are degenerate in the absence of detuning, 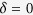, and the system exhibits the charge Kondo effect^43^. The maximum value of the linear conductance is then still given by, 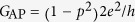. However, there is no plateau any more, instead, 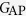 as a function of *δ* exhibits a maximum for 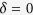 of width 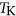, see Fig. 1(a). In the case of 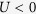, the detuning from particle-hole symmetry point has a similar effect to external magnetic field in the case of the spin Kondo effect^14^. Consequently, when 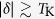, the charge Kondo effect becomes suppressed and the linear conductance suddenly decreases.

In the case of parallel magnetic configuration the situation is completely different. Then, due to the spin-dependence of tunnel couplings, the spin-up and spin-down molecular levels become renormalized in opposite directions, which effectively results in a spin splitting of the levels^40^. This splitting can be tuned by changing the orbital level position, according to the formula^41^,^44^, 
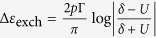
. In fact, this effective exchange field acts in a similar way to external magnetic field^45^,^46^. Consequently, the Kondo effect is present only for 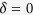 (then 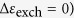, otherwise it becomes suppressed once 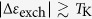. This results in a line for 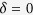 in the *δ *− *U* plane along which the Kondo effect is present, see Fig. 1(b). The width of this line is determined by the Kondo temperature 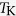. Although the maximum in 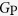 occurs for 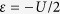 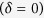, the origin of the Kondo state destruction, depending of the sign of *U*, is different. In the spin Kondo effect regime 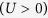 it is the exchange field that breaks the spin degeneracy and leads to the conductance suppression when 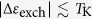. On the other hand, in the charge Kondo effect regime 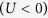 the detuning from particle-hole symmetry point is directly responsible for the conductance drop, which occurs once 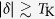. We note that the exchange field is also present when 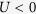 (and 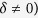, however, its influence is much smaller since it only modifies the energies of excited virtual states responsible for the Kondo effect. Because it does not affect the ground state two-fold degeneracy, it cannot quench the Kondo phenomenon. At this point, we also would like to notice that if the junction is asymmetric, the transport properties in the antiparallel configuration become qualitatively similar to those in the parallel configuration with different values of effective tunnel coupling and effective spin polarization.

The difference between the two magnetic configurations is directly visible in the TMR, which is shown in Fig. 1(c). When electron transport is mainly due to elastic cotunneling processes, the TMR is then given by the Julliere value^47^, 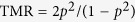, which characterizes a single tunnel junction^48^. This transport regime is in fact present in larger part of the *δ *− *U* parameter space presented in the figure. Nevertheless, the most interesting behavior of the TMR as a function of level position can be observed around 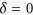, where the difference between the two magnetic configurations is enhanced due to the presence of the exchange field. In the case of positive-*U* one observes a relatively broad region of negative TMR, while in the case of negative-*U*, the TMR becomes suppressed around 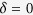. Because the behavior of the linear conductance and the TMR in the Kondo regime in the case of repulsive Coulomb correlations have already been studied elsewhere^41^, here let us focus on the attractive-*U* case in greater detail.

**Figure 2:**
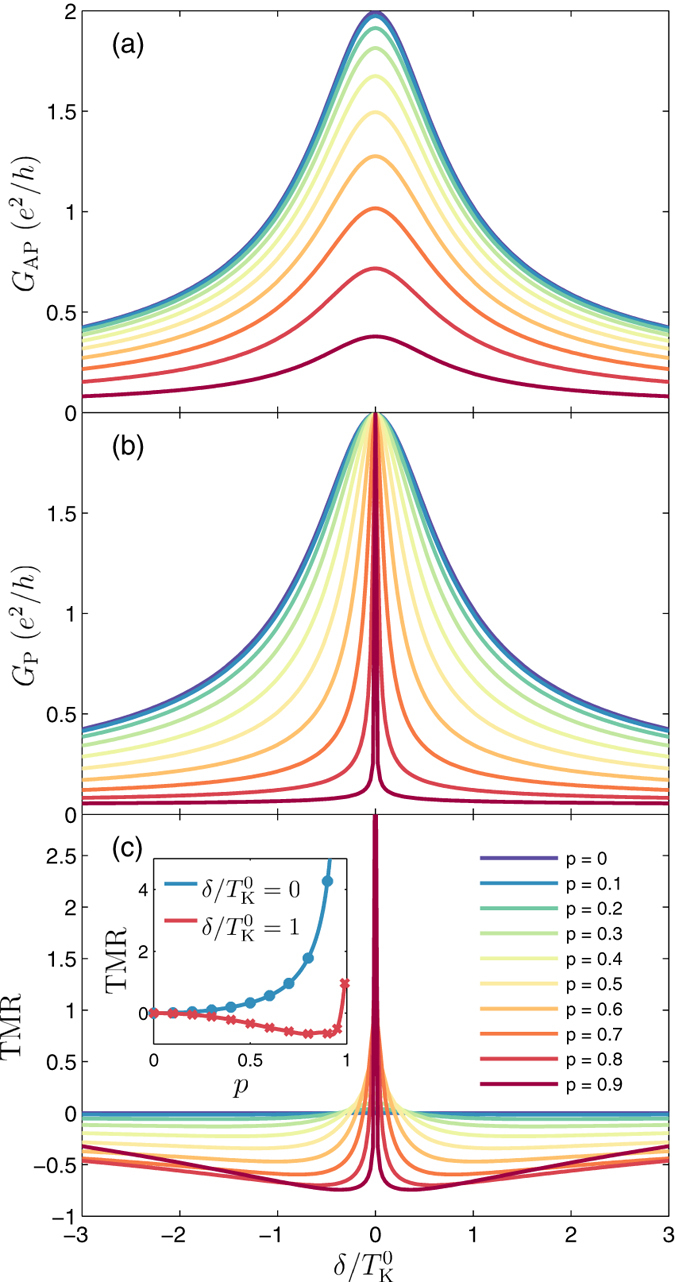
Detuning dependence of magnetoresistive properties. The linear conductance in (**a**) the antiparallel and (**b**) parallel magnetic configuration, and (**c**) the TMR as a function of 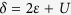 for different spin polarization of the leads *p*, and for 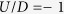, 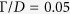 and 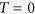. The inset in (**c**) shows the TMR for 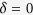 and 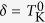 as a function of spin polarization *p*. 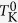 denotes the Kondo temperature in the case of 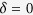 and 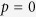.

Figure 2 shows the linear conductance and the TMR as a function of detunig *δ* around the Kondo resonance calculated for different spin polarizations of ferromagnetic contacts. The Kondo temperature for nonmagnetic system 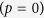 and parameters assumed in Fig. 2 obtained from the half-width at half maximum of the spectral function is equal to, 
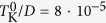
. In both magnetic configurations, the Kondo resonance is clearly visible for 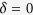. With increasing spin polarization, however, one observes completely different behavior depending on magnetic configuration. In the antiparallel alignment of leads’ magnetic moments the height of maximum decreases with increasing 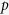 according to 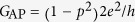, while the relative width of the maximum does not change, see Fig. 2(a). On the other hand, in the case of parallel alignment of leads’ magnetizations, the height of the Kondo resonance is given by 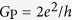, while its width decreases with increasing *p*, see Fig. 2(b). The related behavior of the TMR is shown in Fig. 2(c). Whereas for 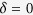, the TMR is given by, 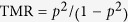, for values of *δ* considered in the figure and for 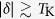, the TMR becomes negative. This is simply related to the fact that the suppression of 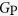 occurs for smaller value of *p* than that of 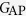, such that 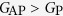 and 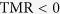. Note, however, that when *p* approaches unity, i.e. for half-metallic leads, the conductance in the antiparallel configuration tends to zero, while the conductance in the parallel configuration is finite, although there is no Kondo effect since the leads provide only one spin species. Consequently, one has 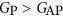 and the TMR becomes positive. This behavior is presented in the inset to Fig. 2(c). In the case of 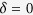, the TMR grows monotonically with *p* and is well-described by 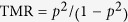, while for 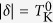, this dependence is not monotonic.

**Figure 3:**
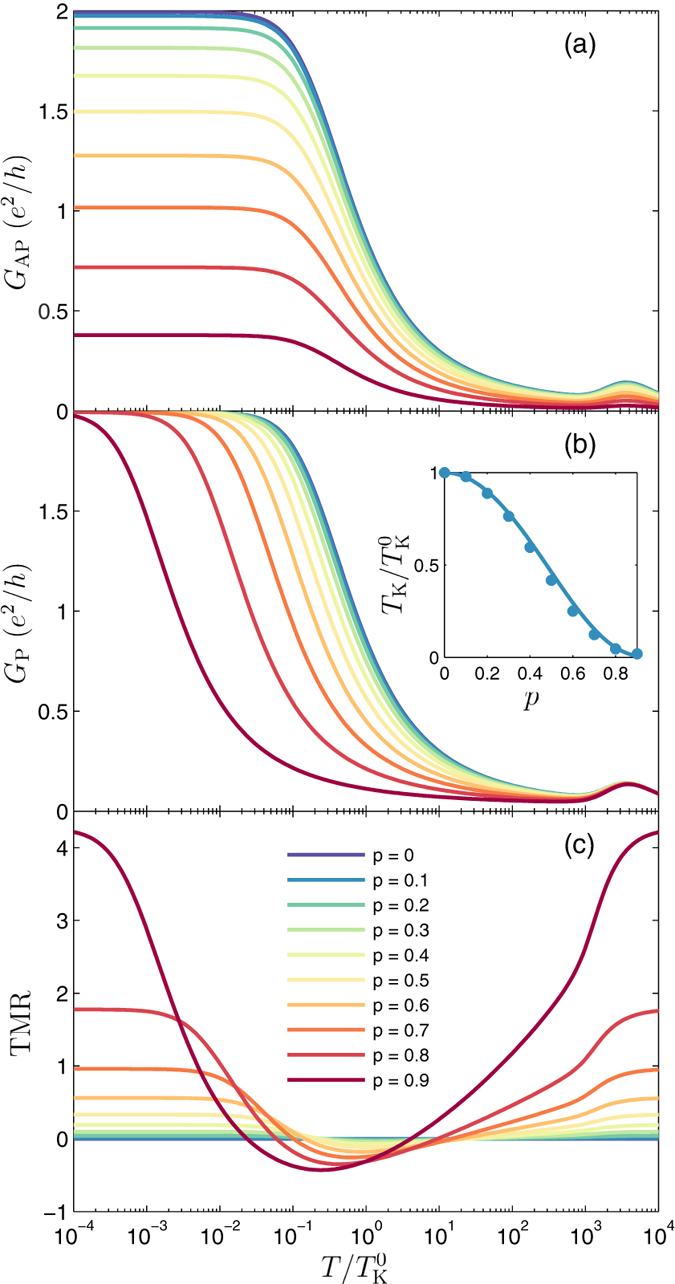
Temperature dependence of magnetoresistive properties. The temperature dependence of the linear conductance in (**a**) the antiparallel and (**b**) parallel magnetic configuration, and (**c**) the TMR calculated for different spin polarization of the leads *p* and for 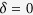. The inset shows the Kondo temperature in the parallel configuration extracted from temperature dependence of 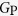 (dots) and that obtained from Eq. (1) (solid line). The other parameters are the same as in Fig. 2.

To demonstrate the influence of leads’ spin polarization, we also analyze the temperature dependence of transport properties. Figure 3 presents 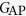 and 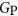, as well as the TMR as a function of temperature for different values of *p* in the case of 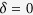. The suppression of 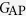 at 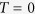 with increasing *p* is clearly visible, see Fig. 3(a). Moreover, the Kondo temperature, defined as temperature at which 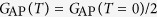, does not depend on *p*. On the other hand, in the parallel configuration an opposite situation is observed, while 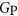 at zero temperature does not depend on *p*, 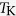 decreases with increasing spin polarization, see Fig. 3(b), according to the formula^49^


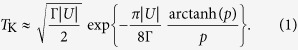


Note that this expression is similar to that in the case of positive-*U* Anderson model in the absence of exchange field-induced splitting^40^. The inset in Fig. 3 compares the numerically calculated 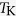 extracted from the temperature dependence of 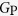 with the formula (1). The agreement is indeed very good. Finally, the temperature dependence of the TMR is depicted in Fig. 3(c). It can be clearly seen that in the low-temperature 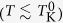 and high-temperature 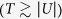 regimes the TMR takes the value 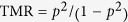, while in the intermediate regime, 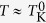, the TMR becomes negative, see Fig. 3(c).

### Spin thermoelectric properties

**Figure 4:**
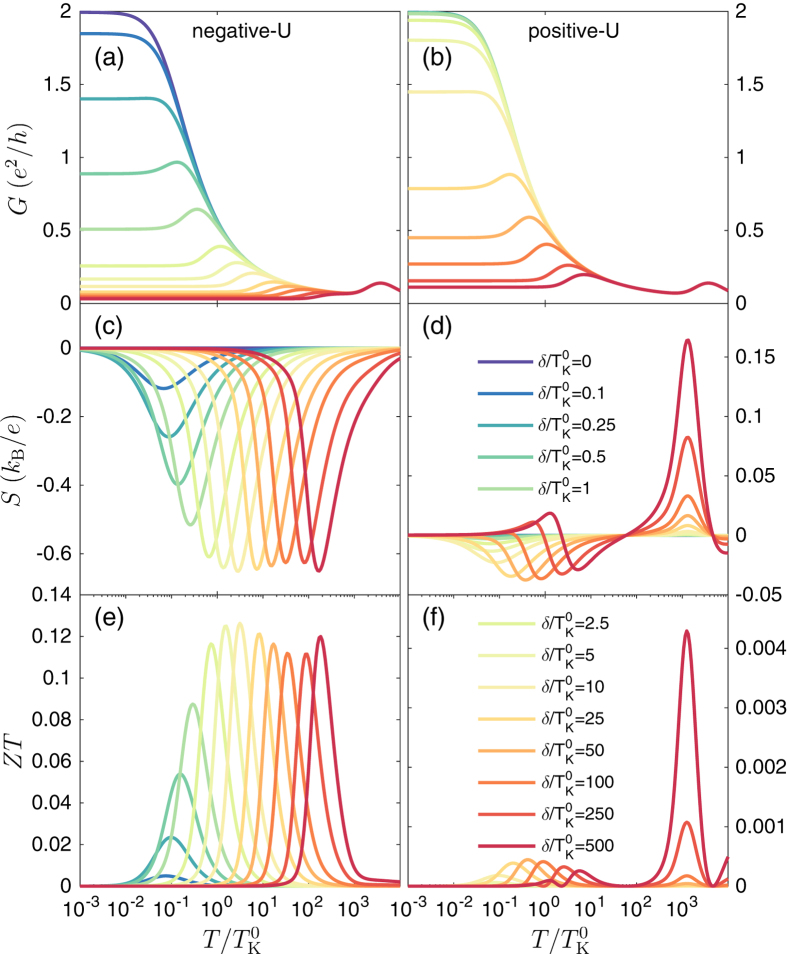
Thermoelectric properties in the absence of spin accumulation. The temperature dependence of [(**a**,**b**)] the linear conductance *G*, [(**c**,**d**)] the Seebeck coefficient *S*, and [(**e**,**f**)] the figure of merit *ZT* in the parallel magnetic configuration for the case of no spin accumulation in the leads. The data is calculated for different orbital level detuning *δ*, as indicated. The left column corresponds to the case of negative−*U*, 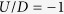, while the right column presents the data in the case of 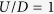, for comparison. The other parameters are the same as in Fig. 2 with 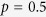.

When the spin relaxation in ferromagnetic leads is fast, there is no spin accumulation and thermoelectric coefficients can be calculated in a similar way as for nonmagnetic systems, i.e. by using equations (5)–(7), 115, 117. Figure 4 presents the temperature dependence of the linear conductance, the Seebeck coefficient and figure of merit in the case of parallel magnetic configuration. The left column corresponds to the case of attractive correlations, while, for comparison, in the right column we also show the results obtained in the case of repulsive correlations. In both cases the absolute value of the Coulomb correlation strength is the same and the curves are calculated for different values of detuning *δ* from the particle-hole symmetry point, as indicated. Although at first sight the temperature dependence of the linear conductance is similar in both cases, there are some differences, as mentioned previously. Finite detuning generally causes the suppression of the Kondo resonance. This suppression takes place faster in the case of negative-*U*, which is related to the fact that the Kondo effect becomes then quenched once 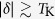. On the other hand, for the positive-*U* case, the Kondo peak gets suppressed only when 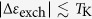. Since for relevant parameters 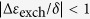, one needs larger *δ* to suppress the Kondo resonance in the case of repulsive correlations.

Contrary to electrical conductance, the thermoelectric properties are clearly different in both cases. While for positive-*U* case the Seebeck coefficient is rather small unless large detuning is induced, for negative-*U* case even relatively small detunings produce considerable thermopower, cf. Figure. 4(c,d). Moreover, the Seebeck coefficient for attractive correlations is always negative, which indicates the role of hole processes in transport (the sign of *S* will change when changing the sign of detuning *δ*), and it exhibits a peak at temperatures approximately corresponding to *δ*. This is just opposite to *S* in the case of 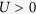 where thermopower exhibits sign changes and is rather small^35^. The difference between those two cases is clearly reflected in the temperature dependence of the figure of merit, which is shown in Fig. 4(e,f). In the case of 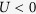, 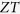 becomes greatly enhanced and exhibits maximum for temperatures corresponding to detuning 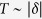. Consequently, at fixed temperature, one can obtain an enhancement of the figure of merit as compared to the case of 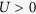 by tuning the orbital level position, which can be done with a gate voltage.

**Figure 5:**
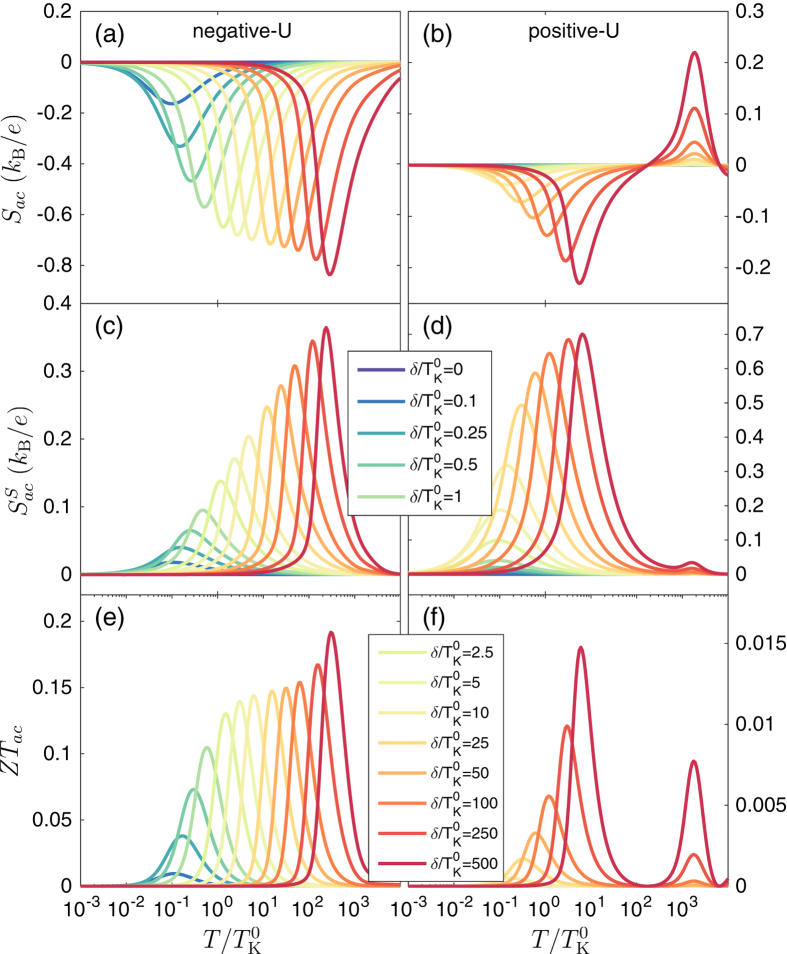
Spin-caloritronic properties in the presence of spin accumulation. The temperature dependence of spin thermoelectric coefficients in the case of finite spin accumulation in the leads: [(**a**,**b**)] the Seebeck coefficient 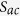, [(**c**,**d**)] the spin Seebeck coefficient 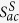, and [(**e**,**f**)] the figure of merit 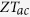 in the parallel magnetic configuration calculated for different orbital level detunings *δ*, as indicated. The left (right) column shows the data obtained for negative−*U*, 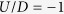 (positive−*U*, 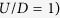. The other parameters are the same as in Fig. 2 with *p* = 0.5.

The spin caloritronic properties in the presence of spin accumulation in the leads (no spin relaxation) are shown in Fig. 5. Again, the left column corresponds to the case of attractive correlations, while the left column presents the case of repulsive correlations, for comparison. Similar to the case of fast spin relaxation shown in Fig. 4, the Seebeck coefficient 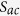 exhibits a comparable dependence on temperature - it is negative and features a peak for 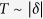, see Fig. 5(a). On the other hand, for assumed parameters the spin Seebeck coefficient 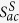 is always positive [Fig. 5(c)], which indicates that the spin-up electrons are the majority ones, cf. Eq. (10). Now, by closer inspection of Fig. 5(c,d) one can see a larger spin thermopower in the case of positive-*U* case. This is however only the case for relatively large detunings, such that the Kondo resonance becomes suppressed by the exchange field, which happens for 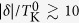. For smaller detunings, the spin thermopower is larger in the case of attractive correlations. Furthermore, as far as the figure of merit is concerned, now we again find a considerable enhancement of 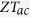 in the case of attractive correlations compared to the opposite case, cf. Fig. 5(e,f). As can be seen in the figure, for molecular junctions with 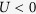, there is a boost in the figure of merit for temperatures corresponding to the detuning from the particle-hole symmetry point, 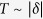. Finally, we would like to mention that, although the obtained values of 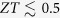 are not as high as in systems exhibiting quantum interference effects^10^, the observed enhancement of 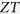 due to attractive correlations is still considerable. Moreover, further enhancement of the spin caloritronic properties can be obtained by using ferromagnets with larger spin polarization.

## Discussion

In conclusion, we have studied the spin caloritronic properties of a correlated molecular junction involving a single orbital level with attractive Coulomb correlations coupled to external ferromagnetic leads. First, we have demonstrated how the emergence of attractive correlations affects the general magnetoresistive transport properties of the system. We have found a particular dependence of the Kondo peak on detuning from particle-hole symmetry point and on magnetic configuration of the device. As far as spin-thermoelectric properties are concerned, we have shown that in molecules with attractive correlations the Seebeck coefficient can be enhanced, which results in a considerable enlargement of the thermoelectric figure of merit. Our findings shed a new light on spin-thermoelectric transport properties of nanoscale objects with attractive Coulomb correlations attached to ferromagnetic contacts and may be of importance for spin caloritronics and molecular spintronics.

## Methods

The Hamiltonian of the considered system is given by the Anderson model^36^


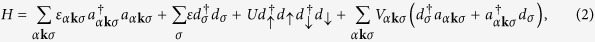


where the first two terms describe noninteracting electrons in the leads and in the molecule, respectively. Here, 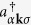 creates an electron of spin *σ* momentum **k** and energy 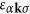 in the left 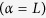 or right 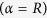 lead, while 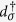 creates a spin-*σ* electron in the orbital level and *ε* is the corresponding energy. The third term of the Hamiltonian takes into account the effective Coulomb correlations in the molecule denoted by *U*, while the last term describes tunneling processes between the molecule and the leads, with 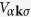 being the respective tunnel matrix element. The strength of the coupling between the orbital level and external leads is given by, 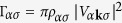, where 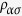 is the spin-dependent density of states of lead *α*, which is assumed to be flat.

The thermoelectric transport coefficients are calculated by using the numerical renormalization group method. The respective quantities can be expressed in terms of the Onsager integrals^50^,^51^


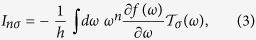


with 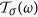 denoting the transmission coefficient and 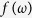 being the Fermi-Dirac distribution function. The transmission coefficient can be related to the spectral function of the orbital level, 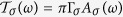, which we calculate by NRG. The linear conductance *G* is then simply given by


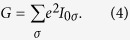


On the other hand, if there is a temperature gradient *δT* applied between the left and right leads, which induces a voltage drop *δV*, the Seebeck coefficient *S* can be found from


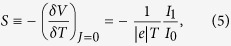


where 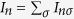, on the condition of vanishing of the charge current 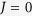. Then, the thermoelectric figure of merit *ZT* can be defined as


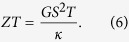


Here, 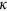 is the thermal conductance, which can be calculated from


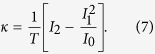


The above formulas were obtained assuming that the voltage drop induced by the temperature gradient is the same for each spin direction. However, if the spin relaxation rate in ferromagnetic leads is sufficiently slow, a spin-dependent voltage drop 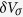 can be induced, giving rise to the spin accumulation, 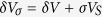, where 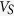 is the spin voltage^28^. Then, the spin-dependent Seebeck coefficient in the presence of spin accumulation can be defined as follows,


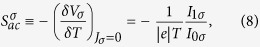


where 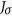 is the current flowing in the spin channel *σ*. The thermopower and the spin thermopower are then given by


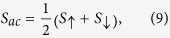



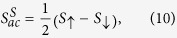


while the figure of merit can be found from


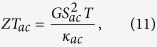


where


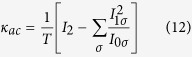


is the thermal conductance in the case when spin accumulation occurs in the leads.

## Additional Information

**How to cite this article**: Weymann, I. Boosting spin-caloritronic effects by attractive correlations in molecular junctions. *Sci. Rep.* **6**, 19236; doi: 10.1038/srep19236 (2016).
